# Head and Eye Movements During Pedestrian Crossing in Patients with Visual Impairment: A Virtual Reality Eye Tracking Study

**DOI:** 10.3390/jemr18050055

**Published:** 2025-10-15

**Authors:** Mark Mervic, Ema Grašič, Polona Jaki Mekjavić, Nataša Vidovič Valentinčič, Ana Fakin

**Affiliations:** 1 Eye Hospital, University Medical Centre Ljubljana, Grablovičeva 46, 1000 Ljubljana, Slovenia; markmervic4@gmail.com (M.M.); ema.grasic1@gmail.com (E.G.); polona.jakimekjavic@kclj.si (P.J.M.); natasa.vidovic@kclj.si (N.V.V.); 2 Faculty of Medicine, University of Ljubljana, Vrazov Trg 2, 1000 Ljubljana, Slovenia

**Keywords:** visual impairment, virtual reality, eye tracking, eye movement, pedestrian crossing

## Abstract

Real-world navigation depends on coordinated head–eye behaviour that standard tests of visual function miss. We investigated how visual impairment affects traffic navigation, whether behaviour differs by visual impairment type, and whether this functional grouping better explains performance than WHO categorisation. Using a virtual reality (VR) headset with integrated head and eye tracking, we evaluated detection of moving cars and safe road-crossing opportunities in 40 patients with central, peripheral, or combined visual impairment and 19 controls. Only two patients with a combination of very low visual acuity and severely constricted visual fields failed both visual tasks. Overall, patients identified safe-crossing intervals 1.3–1.5 s later than controls (*p* ≤ 0.01). Head-eye movement profiles diverged by visual impairment: patients with central impairment showed shorter, more frequent saccades (*p* < 0.05); patients with peripheral impairment showed exploratory behaviour similar to controls; while patients with combined impairment executed fewer microsaccades (*p* < 0.05), reduced total macrosaccade amplitude (*p* < 0.05), and fewer head turns (*p* < 0.05). Classification by impairment type explained behaviour better than WHO categorisation. These findings challenge acuity/field-based classifications and support integrating functional metrics into risk stratification and targeted rehabilitation, with VR providing a safe, scalable assessment tool.

## 1. Introduction

The number of individuals with visual impairment is estimated at 553 million people and is increasing globally, making it a significant public health challenge for the ageing population [1,2]. The World Health Organization (WHO) classifies visual impairment into different categories based on standardized measures of visual acuity (VA) and visual field (VF) [3]. However, these classical monocular measures do not always reflect how individuals function in complex day to day activities, such as reading, navigating in supermarkets, or walking and driving in traffic [4,5].

To address this gap, functional assessment has gained traction. Currently established approaches include mobility courses in real and virtual environments, driving simulators, virtual reality (VR)-based tests with integrated eye tracking for face recognition, and reading and other complex activities [4,6,7,8]. For example, a recent study validated the Mobility Standardized Test in both real and virtual environments for patients with rod–cone dystrophies, supporting VR as a controlled yet ecologically meaningful testbed for mobility outcomes [6]. Driving-simulator work shows how visual impairment or its simulation degrades hazard detection and scanning, highlighting sensitivity to age and distraction [7]. Beyond locomotion, VR with integrated eye tracking has been used to quantify oculomotor behaviour in central visual impairment during reading and to characterize face-viewing strategies within immersive head-mounted displays [4,8].

Contemporary reviews converge on the same message: VR systems paired with eye tracking sensors can provide precise, standardized measurements of oculomotor behaviour, e.g., saccadic movements, microsaccades, fixations, smooth pursuit, pupil variables, as well as head movements under immersive conditions [9,10,11]. This approach offers valuable insights into the behavioural consequences and compensatory mechanisms associated with peripheral or central visual impairment, extending beyond traditional clinical measures [5,10,11]. VR further offers automated, standardized, low-cost, and positive user experience tests and has been successfully applied in several fields of clinical ophthalmology, improving diagnosis, screening, and rehabilitation [12,13,14,15,16].

Prior VR work has largely focused on reading or face recognition [4,11,17]. Far fewer studies have examined orientation in traffic and pedestrian crossing decisions; among those that did, many relied on gaze-contingent or optical simulations of VF impairment in normally sighted participants rather than measuring head–eye behaviour in patients.

We therefore aimed to gain a deeper understanding of how various types of visual impairments affect orientation in traffic by using an immersive VR environment integrated with eye tracking. We assessed the perception of moving vehicles and pedestrian crossing decisions in patients with central, peripheral, or combined visual impairment, as well as in healthy controls, to determine how different types of visual impairment affect head–eye movements and task performance and to test whether functional grouping by impairment type better explains behaviour than WHO categorization.

By focusing on pedestrian crossing and orientation in traffic, this study extends VR eye tracking research beyond reading and face recognition to a safety-critical, dynamic behaviour that simultaneously draws on central and peripheral vision. This context can reveal compensatory strategies, or their absence, that static clinical tests may miss. The findings are expected to contribute to the development of more advanced methods for evaluating visual behaviour and functioning beyond standard clinical measures of VA and VF.

## 2. Materials and Methods

### 2.1. Study Subjects

We enrolled consecutive eligible adults with various eye diseases who were overseen by ophthalmologists at the Eye Hospital, University Medical Centre Ljubljana, Slovenia. The final sample comprised 40 patients with visual impairment and 19 normally sighted controls. Our target size was pragmatic/feasibility-based; no a priori power calculation was performed.

Inclusion criteria (patients): categorized by functional loss in the better-seeing eye (BSE) and binocular Esterman VF radius:

- Central visual impairment: BSE uncorrected VA ≤ 0.6 and ≥0.05 (Snellen decimal); binocular VF radius > 50°.
- Peripheral visual impairment: BSE uncorrected VA ≥ 0.4; binocular VF radius ≤ 50° and ≥5°.
- Combined visual impairment: BSE uncorrected VA < 0.4 and ≥0.05; binocular VF radius ≤ 50° and ≥5°.

Thresholds were defined based on WHO visual impairment categories and minimum EU driver-licencing standards (binocular VA ≥ 0.5; binocular horizontal VF ≥ 120° with ≥50° left/right) [18]. We used a 50° VF radius to demarcate predominant peripheral impairment and VA 0.4 (≈0.40 logMAR) to ensure a clear central deficit in the combined group with room for test–retest variability on the Snellen chart [19]. Our lower bounds referenced thresholds of WHO categories 4/5; VA ≥ 0.05 and VF radius ≥ 5°, keeping the study focused on low vision rather than blindness and ensuring participants can meaningfully perform the VR tasks [3]. If the peripheral visual impairment was not concentric, the degree was calculated as the average between temporal and nasal constriction.

We used measurements from the BSE because binocular VA is very closely predicted by the VA of the better eye [20].

Inclusion criteria (controls): binocular uncorrected VA ≥ 0.8 and binocular VF radius ≥ 80.

Exclusion criteria: age < 18 years; spherical equivalent refraction in the BSE ≥ 2.00; since VR testing was performed without refractive correction, there was a difference of >2.00 Snellen decimal between uncorrected VA and BCVA; neurologic disease affected perception. Controls additionally required no history of ocular disease or colour-vision impairment.

### 2.2. Visual Acuity Testing and Visual Field Testing and Imaging

VA was measured using Snellen charts in decimal annotation and converted to logMAR. We considered values measured binocularly or in the BSE.

VF radius was tested binocularly using the Octopus Esterman test (Octopus 900 perimeter, Haag-Streit AG, Köniz, Switzerland). Participants fixated a central target while brief, suprathreshold white stimuli were presented on a predefined grid; they pressed a button when a stimulus was detected. The VF radius was derived as the eccentricity (degrees) of the most peripheral detected locations along each meridian horizontally, then averaged across meridians to yield a mean binocular VF radius. To address reproducibility, we averaged across meridians to stabilize estimates and interpreted borderline values cautiously.

For controls, we documented uncorrected VA and VF radius as meeting the inclusion criteria, since all participants met the entry criteria for normal vision.

Imaging was conducted using fundus autofluorescence (FAF) (Spectralis, Heidelberg Engineering GmbH, Heidelberg, Germany or Optos California Ultra-widefield Retinal Imaging System, Optos plc, Dunfermline, UK) and optical coherence tomography (OCT) (Spectralis, Heidelberg Engineering GmbH, Heidelberg, Germany).

### 2.3. Visual Impairment Categories

Patients were categorized into different subgroups to examine whether any of the measured endpoints correlated with the type and/or degree of visual impairment. The main categorization was determined according to their visual impairment being either central, peripheral, or combined. Additionally, they were assigned the category of visual impairment according to the WHO classification (Table 1) [3].

### 2.4. Cognitive Impairment Screening

Using the highly sensitive MoCA (Montreal Cognitive Assessment, available at https://mocacognition.com, last accessed on 1 August 2025) Blind Test, adapted to exclude the visual component, we screened both the patient and control groups for early signs of cognitive impairment, given the study’s focus on older individuals and the well-established association between cognitive decline and certain ocular diseases (e.g., age-related macular degeneration; AMD). A lower score is associated with a higher risk for cognitive impairment, which could affect traffic orientation testing. We used the resulting score as a covariate (no exclusions were made on the basis of cognitive screening).

### 2.5. Traffic Orientation Testing

#### 2.5.1. VR Apparatus (Headset and Eye Tracking)

Traffic orientation tests were conducted through IC Traffic Simulation v1.0 (Synthesius, Ljubljana, Slovenia) on a VR headset (HP Reverb G2 Omnicept Edition, HP Inc., Palo Alto, CA, USA) and performed binocularly without refractive correction; eye movements were analyzed using IC FUSION v1.2 (Synthesius, Ljubljana, Slovenia).

The headset provides 2160 × 2160 px per eye at 90 Hz with an approximately 114° diagonal field of view and integrates Tobii eye tracking (Tobii AB, Danderyd, Sweden) accessed via the HP Omnicept SDK, yielding binocular gaze at 120 Hz with a point calibration performed between participants. Reported accuracy for Tobii integrations is ~0.5–1.1° within ±20° of primary gaze, with precision declining at larger eccentricities [21,22]. Motion-to-photon latency of modern VR systems is typically in the tens of milliseconds range; given that our key outcomes evolve over seconds, such latencies are negligible at the effect sizes studied and are common to all groups [23].

#### 2.5.2. Tasks and Recordings

**Figure 1:**
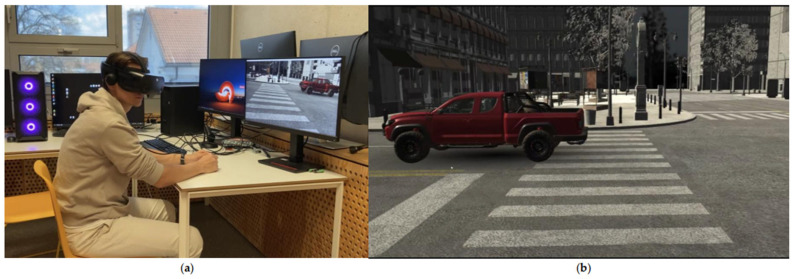
Traffic orientation testing: (**a**) test set-up; (**b**) the participant’s view of the road crossing in virtual reality (VR).

**Figure 2:**
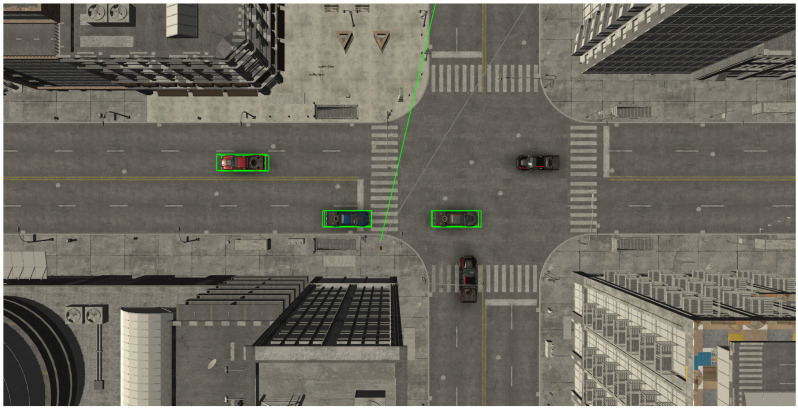
The view of the street with a pedestrian crossing and vehicles of different colours from above. The grey line shows the direction of the head in the VR, and the green line shows the direction of the gaze.

Each participant viewed a 45 s recording that provided a virtual experience of standing as a pedestrian in front of a traffic road. Exactly 10 cars of different colours, among which 3 were red, passed by the scene with two predetermined intervals suitable for crossing the street (16–25 s; 34–45 s). The use of two fixed gaps standardizes task difficulty and measurement across participants. Figure 1 shows the test set-up and the view of the participant, and Figure 2 shows the view of the street from above.

Each participant was shown the scenario twice, performing two different tasks. For the first task, they were instructed to identify the safe road-crossing opportunities, and the time stamp of their verbal response was registered. The number of identified safe road crossings and the time at recognition (crossing time 1; crossing time 2) were analyzed. For the second task, the participants were instructed to count the number of red cars among those driving by. During each viewing, the head and eye movements were recorded automatically. The number of saccades, fixations, and head movements was compared between patients and controls for each task.

For the purpose of this study, the saccades were defined as eye movements with a velocity of >50 °/s [24]. Macrosaccades (often referred to as simply saccades in the literature) were defined as saccades with an amplitude ≥ 1.2° and microsaccades as saccades with an amplitude < 1.2°, which is in concordance with other studies using eye tracking [24].

### 2.6. Statistical Analysis

Statistical analysis was performed in IBM SPSS Statistics v29 (IBM Corp., Armonk, NY, USA). Normality was assessed with the Shapiro–Wilk test.

Descriptive/exploratory analyses: Because most endpoints were non-normal, we report them as median [min–max] and use two-sided non-parametric tests: Mann–Whitney U for two-group comparisons and Kruskal–Wallis for multiple groups (controls vs. central, peripheral, combined; and WHO categories), with Bonferroni-adjusted pairwise contrasts where applicable. Correlations between continuous variables and outcomes were assessed with Spearman’s ρ.

Regression models: For covariate adjustment, we ran separate linear regression models for each outcome (CT1, CT2, and selected eye/head metrics) within each task. To reduce overfitting with our modest *n*, we limited predictors. In all-participant models, predictors were patient/control status, age, and MoCA; for visual-loss-type contrasts, *group* was modelled categorically with controls as reference while adjusting for age and MoCA. Sensitivity analyses included age-only models and within-group correlations. We analyzed WHO categories separately from functional type and did not include both in the same regression model. Unless stated otherwise, tests were two-tailed with α = 0.05, and we report β with *p*-values.

## 3. Results

### 3.1. Participant Demographics

Participants’ demographics are summarized in Table 2 and reported in detail in Supplementary Tables S1 and S2.

### 3.2. Visual Task Performance

#### 3.2.1. Identification of Safe Road Crossing

There was no significant difference in the number of identified safe road crossings between patients and controls (median 2 vs. 2; Mann–Whitney U, *p* > 0.8). Consistent with this ceiling effect, correlations of safe road crossings identified with VA and with VF were small and did not reach significance (VA: ρ = 0.18, *p* = 0.26; VF: ρ = 0.30, *p* = 0.063).

**Figure 3:**
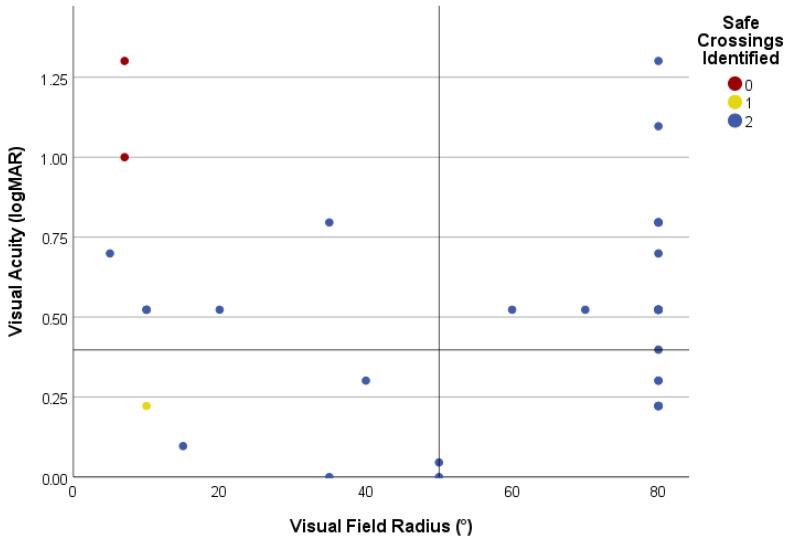
Performance on the safe-crossing task in correlation with visual function. Visual field (VF) radius and visual acuity (VA) are plotted on the x and y axes, respectively. Each patient is presented with a circle, and their colour encodes the number of identified safe-crossing intervals. Note that the number of circles is lower than the number of patients because some with central visual impairment and a normal VF radius overlap. Reference lines mark the study cut-offs used to classify central, peripheral, and combined visual impairment categories.

To investigate the correlation between task success and parameters of visual function in more detail, VA (logMAR) and VF were plotted for each patient (Figure 3). Detection was non-zero across almost the entire VA-VF space. The only two failures (2/59, 3.4%) had a combination of very poor VA (≥1.0 logMAR) with severe VF constriction (≤7°), whereas patients with an isolated defect of either VA or VF of similar severity succeeded in the task.

**Figure 4:**
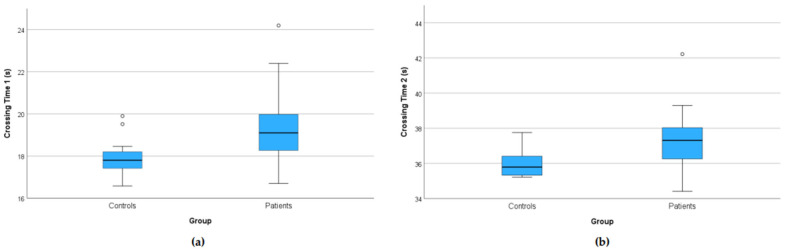
Time points when participants thought it was safe to cross the street. Boxplots show the response times for the first (**a**) and second (**b**) car-free windows during safe crossing. The time range on the y-axis corresponds to the times between 16–25 s and 34–45 s when there were no cars on the road. Patients were delayed at both opportunities relative to controls. Boxes show the interquartile range (25th–75th percentiles); the line is the median; whiskers extend to 1.5 × IQR; circles denote outliers.

Patients identified safe road-crossing opportunities significantly later than controls (Figure 4). The first safe-crossing opportunity was registered at a median 19.1 s by patients vs. 17.8 s by controls (Mann–Whitney U, *p* < 0.01.); the second road-crossing opportunity, registered at a median 37.3 s by patients vs. 35.8 s by controls (*p* = 0.001). In multivariable models adjusting for age and MoCA, patients identified the first safe-crossing opportunity later than controls (β = +1.16 s, *p* < 0.05). The adjusted difference for the second opportunity was directionally similar but did not reach significance (β = +0.55 s, *p* = 0.189).

#### 3.2.2. Identification of Safe Road Crossing by Type of Visual Impairment

**Figure 5:**
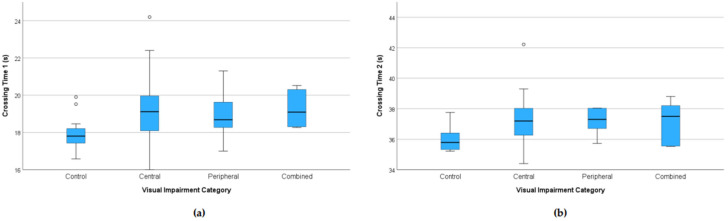
Time points when participants thought it was safe to cross the street. Patients are further categorized by type of visual impairment. Boxplots show the response times for the first (**a**) and second (**b**) car-free windows during safe crossing. The time range on the y-axis corresponds to the times between 16–25 s and 34–45 s when there were no cars on the road. Boxes show the interquartile range (25th–75th percentiles); the line is the median; whiskers extend to 1.5 × IQR; circles denote outliers.

Figure 5 shows identification times of safe-crossing opportunities for controls and patients with central, peripheral, or combined visual impairment. Median identification times for the first safe-crossing interval were 17.80, 19.11, 18.68, and 19.09 s, respectively, while median identification times for the second safe-crossing interval were 35.79, 37.20, 37.30, and 37.50 s, respectively. Pairwise comparisons with Bonferroni correction between controls and patients showed a significant difference for patients with central visual impairment for both crossing times (both padj < 0.05), while other pairwise contrasts did not reach adjusted significance, although delay was similar in all patient subgroups and relatively the longest in patients with combined visual loss.

#### 3.2.3. Car Counting

There was no significant difference in the number of identified red cars between patients and controls (median 3 vs. 3; Mann–Whitney U, *p* = 0.111). Consistent with this ceiling, correlations of cars detected with VA and with VF were small and non-significant (|ρ| ≤ 0.24, *p* > 0.12).

**Figure 6:**
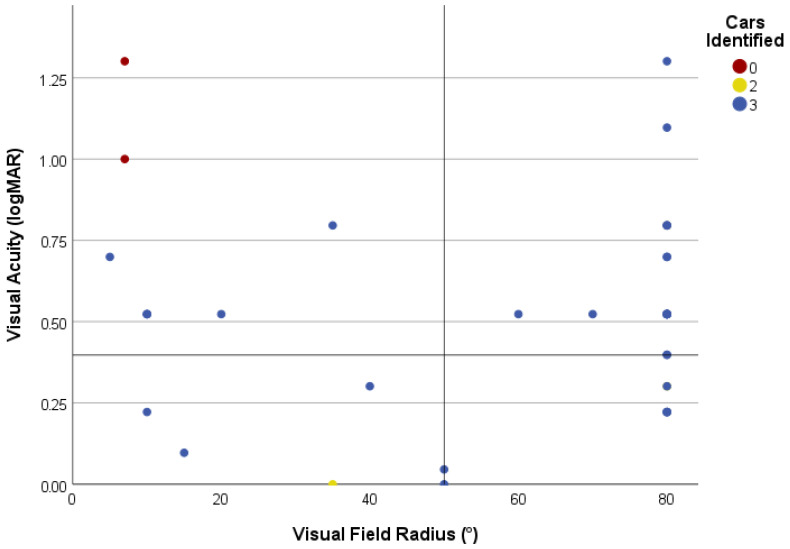
Performance in the car-counting task in correlation with visual function. VF radius and VA are plotted on the x and y axes, respectively; each patient is presented with a circle, and their colour encodes the number of red cars correctly identified. Only two participants with the poorest combined vision (VA ≥ 1.0 logMAR; VF ≤ 7°) failed to identify any cars. Reference lines mark the study cut-offs used to classify central, peripheral, and combined visual impairment categories.

Similarly, as for the identification of safe road crossing, a chart was designed to inspect task success in correlation with the two main parameters of visual function (Figure 6). Notably, the same two patients who failed the safe-crossing task also failed to detect any of the red cars.

### 3.3. Eye and Head Movements

Supplementary Tables S3 and S4 show the summary of eye- and head-movement characteristics of patients and controls during the two visual tasks. Full participant-level data are provided in Supplementary Tables S5–S8.

#### 3.3.1. Head Turns

**Figure 7:**
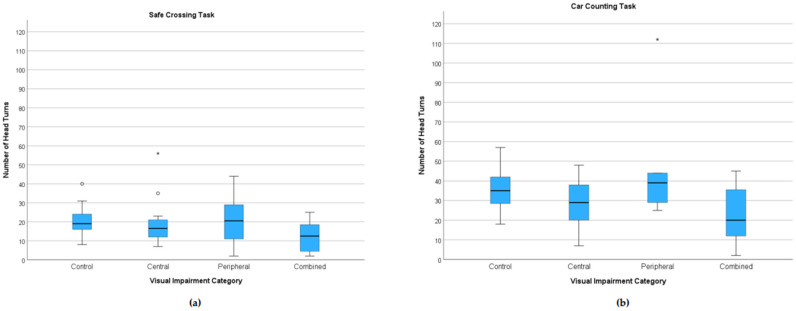
Number of head turns by task and category of visual impairment. Boxplots: (**a**) safe-crossing task; (**b**) car-counting task. In both tasks, patients with combined visual impairment exhibited significantly reduced head turning, while patients with peripheral loss showed comparable medians but broader dispersion during the safe-crossing task. Boxes represent the interquartile range (25th–75th percentiles); the horizontal line is the median; whiskers extend to the extreme values (up to 1.5 × IQR), and circles and stars mark outliers.

Patients with combined visual impairment executed fewer head turns than controls during both the safe-crossing (median 12.5 vs. 19.0; *p* < 0.05) and car-counting task (median 20.0 vs. 35.0; *p* < 0.05). Patients with central visual impairment also exhibited relatively fewer head turns; however, the difference was not significant. The peripheral subgroup showed medians similar to controls (Figure 7). All participants exhibited almost twice as many head turns during the car-counting task in comparison to the safe-crossing task. In models adjusting for age and MoCA, combined vs. control difference during the car-counting task remained significant (β = −14.47, *p* < 0.05), whereas the reduction during safe crossing was directionally similar but did not reach significance (β = −7.50, *p* = 0.102).

#### 3.3.2. Number of Macro- and Microsaccades

**Figure 8:**
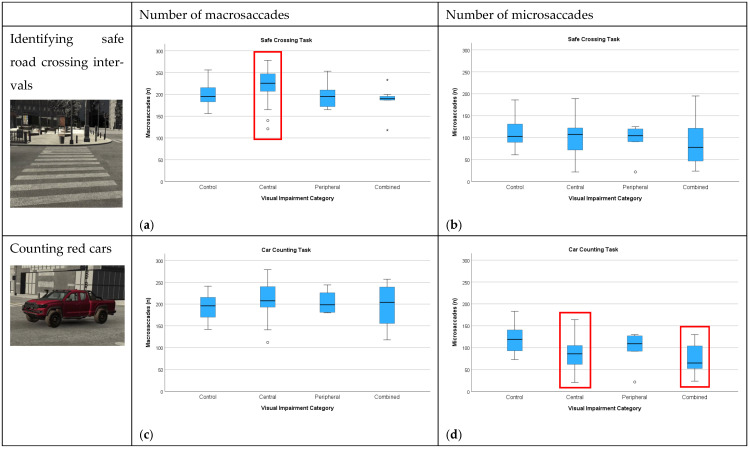
Number of macro- and microsaccades by task and category of visual impairment. Safe-crossing task: (**a**) patients with central visual impairment made more macrosaccades than controls, whereas (**b**) microsaccade counts did not differ significantly between subgroups. Car-counting task: (**d**) microsaccade counts were lower in patients with central and combined visual impairment, while (**c**) macrosaccade counts did not differ across subgroups. Boxes show the interquartile range (25th–75th percentiles); the line is the median; whiskers extend to 1.5 × IQR; circles and stars denote outliers. Red rectangles denote significant differences from the control group.

The number of macro- and microsaccades executed during different visual tasks is presented with boxplot charts for each subgroup in Figure 8. During the safe-crossing task, patients with central visual impairment executed significantly more macrosaccades than controls (median 226 vs. 195; *p* < 0.05), while other patient subgroups did not show notable differences from controls. Accordingly, since fixation can be defined as a relative pause between two macrosaccades, patients with central visual impairment had a significantly higher number of fixations, which were also significantly shorter than those of controls (mean 0.200 s vs. 0.231 s, *p* < 0.05). Patients with combined visual impairment had a relatively lower median number of microsaccades; however, this difference was not significant. In models adjusting for age and MoCA, group differences in the number of macrosaccades and average fixation duration did not persist after adjustment (both *p* > 0.25).

During the car-counting task, there was no significant difference in the number of macrosaccades across different patient subgroups, whereas patients with central and combined visual impairment executed significantly fewer microsaccades than controls (86 and 65 vs. 119, respectively; *p* < 0.05). In models adjusting for age and MoCA, the difference between central and combined vs. controls remained significant (β = −36.1, *p* < 0.01; β = −43.6, *p* < 0.05).

Note that there was an increase in the number of microsaccades during car counting in comparison to safe crossing for controls but not other subgroups (rather the reverse).

#### 3.3.3. Saccade Kinematics

Saccade kinematics were described by amplitude, duration, and velocity and are reported in Supplementary Tables S3 and S4. Parameters that showed significant differences between controls and patients and have not yet been presented are reviewed below.

##### Total Macrosaccade Amplitude (°)

**Figure 9:**
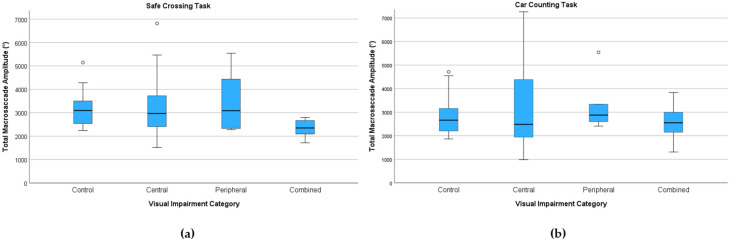
Total macrosaccade amplitude (°) by task and visual impairment category. Boxplots: (**a**) safe-crossing task; (**b**) car-counting task. Boxes show the interquartile range (25th–75th percentiles); the line is the median; whiskers extend to 1.5 × IQR; circles denote outliers.

For the safe-crossing task, the median total macrosaccade amplitude was lowest for patients with combined visual impairment (2353 °) relative to controls (3098 °), central (2970 °), and peripheral (3089 °) (Figure 9). Pairwise comparisons versus controls indicated a significant reduction for the combined group (*p* < 0.01), which persisted in models adjusted for age and MoCA (β = −1156 °, *p* < 0.05).

For the car-counting task, medians were closer (control 2663 °, central 2485 °, peripheral 2877 °, combined 2552 °) and not statistically significant.

##### Average Microsaccade Velocity (°/s)

In the safe-crossing task, patients showed an elevation of average microsaccade velocity vs. controls, most prominently in those with central and combined visual impairment (median velocities: control 115.3, central 118.8, peripheral 117.0, combined 124.3 °/s) (Supplementary Figure S1). Planned two-sided Mann–Whitney tests were significant only for the central group (*p* < 0.05). In adjusted models for age and MoCA, the difference was significant for central and combined vs. controls (β = +10.0 °/s, *p* < 0.05; β = +21.8 °/s, *p* < 0.001).

For the car-counting task, the pattern was similar but not statistically significant (medians: 116.7, 120.1, 117.7, 124.6; Kruskal–Wallis *p* = 0.147).

#### 3.3.4. Visual Task Performance by WHO Visual Impairment Category (Exploratory)

In addition to the analysis of differences between the patients with central, peripheral, and combined visual impairment, we also examined outcomes across the WHO visual impairment categories (0–4) (controls reported separately). As for CT1/CT2 and the number of red cars identified, no monotonic trend emerged (|ρ| ≤ 0.18, *p* ≥ 0.30). Oculomotor metrics showed a weak pattern: in the safe-crossing task, patients within WHO low-vision category 3 had a reduced ‘macro’ output: lower average macrosaccade velocity (*p* < 0.05 vs. patients not yet categorized by WHO; *p* < 0.01 vs. controls) and smaller total macrosaccade amplitude (Kruskal–Wallis across WHO categories *p* < 0.05; vs. controls *p* < 0.001).

In contrast, earlier categories diverged from controls mainly in their ‘micro’ output during the car-counting task. Patients withing WHO low-vision category 1 showed fewer (*p* < 0.01 vs. controls) and smaller amplitude (*p* < 0.05 vs. controls) microsaccades; patients within WHO low-vision category 2 showed similar trends (fewer microsaccades, *p* < 0.05 vs. controls), and even patients not yet categorized by WHO differed slightly (higher average microsaccade velocity, *p* < 0.05 vs. controls) (Supplementary Figures S2–S4).

## 4. Discussion

This study used a novel system combining a VR headset equipped with eye tracking sensors to determine characteristics of head and eye movements of patients with visual impairment during different visual tasks performed in a realistic 3D environment.

### 4.1. Visual Task Performance

The majority of patients demonstrated good ability to identify the predefined visual targets, i.e., empty road crossings and red cars, which suggests that patients with mild-to-moderate visual impairment have fairly good orientation in the road traffic environment. A key observation of the present study is the disproportionately severe impact of combined visual impairment. The two patients with a combination of a very poor VA (≤0.1) and VF (≤7°) were the only ones who failed to detect any safe crossings or any red cars (Figure 3 and Figure 6). Neither VA nor VF alone predicted failure; however, their combination did, implying a loss of compensatory potential rather than a mere sum of deficits. Prior VR-based findings likewise show that simulated impairment of central and peripheral vision produces different impairments in naturalistic search behaviour. Central loss mainly slows target verification, whereas peripheral loss mainly disrupts navigation, implying that simultaneous impairments could remove complementary sources of information and compensation [5].

Although the majority of patients were able to identify the visual targets, they needed significantly longer than controls to decide that it was safe to cross the road, ranging between 1.3 and 1.5 s for the two crossing intervals. For context, experimental street-crossing tasks report accepted crossings with a safety margin between 0 and 1.5 s as “tight fits”, and the shares of both tight fits and unsafe decisions rise with vehicle speed [25]. In our cohort, a 1.3–1.5 s delay is large enough to push some choices into the tight-fit range and therefore elevate risk in faster, denser traffic. Moreover, older pedestrians often fail to compensate fully for slowed walking speed when selecting gaps [26]. This combination, therefore, plausibly makes moving around (e.g., crossing the street) more time-consuming and potentially more dangerous for visually impaired and older pedestrians, especially if the visual impairment also slows down walking speed [27].

After pairwise comparisons between subgroups, significance was retained for patients with central visual impairment. This is in agreement with a previous study that showed that when central scotomas are simulated in pedestrians within a VR roundabout, people wait longer and choose later, larger traffic gaps before initiating a crossing [28]. The delays of patients with peripheral and combined visual impairment were directionally similar (relatively the longest in the combined group), but non-significant (Figure 5), most likely due to the smaller number of patients. A larger study is needed to confirm the findings.

### 4.2. Head and Eye Movement Analysis

Head and eye movement behaviour was shaped by the visual task and type of visual impairment.

Patients with combined visual impairment displayed the most concerning profile: the lowest total amplitude of macrosaccades during the safe-crossing task and the fewest head turns across both tasks, indicating severe under-scanning. Several constraints likely converge. First, reduced VA slows visual search, especially when targets must be distinguished by their shape or size, precisely the type of discrimination our scenes required [29]. Second, bilateral vision loss at and around the fovea degrades saccade quality. In AMD patients, many saccades are programmed toward an eccentric preferred retinal locus rather than the fovea, so they are often misdirected or curved and show lower peak velocity and longer duration, undermining efficient large gaze shifts [30]. At the same time, peripheral VF impairment compromises the very signals that usually trigger exploratory saccades—peripheral cues that normally guide the next fixation—often yielding suboptimal compensation during naturalistic search [31]. Taken together, our data and prior evidence suggest that when central and peripheral deficits co-occur, patients default to shorter, more hesitant head and eye movements, leaving substantial parts of the scene uninspected within the available time.

Patients with central visual impairment made significantly more macrosaccades and accordingly had more and shorter fixations in comparison to controls during the safe-crossing task. This is consistent with previously described eccentric-viewing strategies in patients with AMD [30]. Bilateral vision loss at and around the fovea not only impairs VA but also introduces challenges for eye movements due to the loss of the foveal oculomotor reference, resulting in curved scan paths of the eyes and requiring multiple small saccades to reach the target [32]. By contrast, patients with peripheral visual impairment showed saccade numbers similar to controls, with a trend toward more frequent head turns associated with narrower VF in the safe-crossing task (Supplementary Figure S5). This pattern is consistent with on-road and driving-simulator studies showing that participants with binocular VF impairment or hemianopia compensate by executing more head turns [33,34]. Additionally, during naturalistic visual search, peripheral VF impairment appears to alter the direction of gaze, tending to reduce movements into less sensitive regions and altering saccade patterns, rather than simply increasing the number of saccades [31].

In the car-counting task, however, patients with central and combined visual impairment showed fewer microsaccades and a shorter total duration of microsaccades than controls. This finding is consistent with evidence that microsaccades depend on a foveal “anchor”. When the fovea is engaged, microsaccades are generated easily, whereas peripheral stimulation alone is insufficient to produce normal microsaccades, with rates dropping when foveal input is removed [35]. In healthy observers, imposing a simulated central scotoma produces the same effect—microsaccade frequency drops, and the reduction becomes larger as the scotoma grows [36]. Cognitive load likely compounds the effect: microsaccade rates reliably drop as attentional demand increases, especially when attention must be concentrated at fixation [37]. In contrast, the safe-crossing scenario pulls gaze into broad head–eye exploratory sweeps toward peripheral traffic. In tasks like this, large exploratory saccades dominate, and microsaccades, which mainly arise during brief fixation pauses, become relatively infrequent [38]. Consistent with this pattern, our data show that between-group differences in microsaccades are small.

Note that, although statistically significant, these comparisons, especially for peripheral and combined subgroups, which were relatively small, should be regarded as exploratory signals that warrant confirmation in larger, balanced cohorts.

### 4.3. Categorization of Visual Impairment

While individual variability exists, these group patterns argue for moving assessment beyond charts toward functional vision characterization. The WHO visual impairment categories based on VA and/or VF are valuable for epidemiology and eligibility but mix distinct phenotypes.

In our study, 2/40 (5%) patients failed to detect all visual targets (Figure 3 and Figure 6); both had very low VA (≤0.1 Snellen) and severe VF constriction (≤7°) and would fall into WHO category 3 on either criterion. Yet 5/40 (12.5%) patients who were also classified as WHO category 3 (by either low VA or decreased VF, but not both at the same time), and even 1 participant classified as WHO category 4 (due to VF radius = 5° but with relatively preserved VA = 0.20), successfully identified at least one car and one safe crossing, suggesting that the combination of severe central and peripheral loss is disproportionately more debilitating than an isolated impairment within the same category. Consistent with this, stratifying by type of visual impairment better explained task performance and eye-movement patterns in our study. These findings should be regarded as exploratory, given small, imbalanced groups and warrant confirmation in larger, balanced cohorts.

### 4.4. Implications for Functional Classification and the Potential of VR Assessment

Our findings argue for supplementing categorical severity labels (e.g., WHO/ICD) with functional descriptors that specify whether loss is central, peripheral, or combined. People with comparable VA or VF labels can diverge meaningfully in everyday performance: mobility safety often depends on the VF even when VA is similar, whereas reading is constrained primarily by central vision regardless of VF [39,40,41]. The pattern that combines central and peripheral impairment is particularly disabling, identifying a phenotype that may warrant closer monitoring and targeted support. Clinically, brief functional tests alongside charts may aid risk assessment and referral.

Rehabilitation priorities may also be phenotype-specific. For peripheral VF impairment, structured scanning protocols that reinforce horizontal exploration and deliberate head turns are plausible; a randomized study of exploratory training reported improvements in mobility and oculomotor measures in retinitis pigmentosa patients [42]. For central visual impairment, VR provides a safe setting to practise gap perception during street-crossing, with effects comparable to real-world instruction [43]. For combined visual impairment, where severe under-scanning was observed, a controlled, multicomponent approach seems prudent: gradual increase in task difficulty in VR, explicit coaching of eye-movement and head-turning strategies, and an emphasis on safety before speed. Because visual and cognitive function interact in older adults, routine cognitive screening and pacing may be warranted [44]. All such applications should be treated as testable hypotheses for future work rather than established standards.

Methodologically, head-mounted VR with integrated eye tracking proved valuable for functional assessment. It delivered a realistic yet safe and standardized environment, with automated, high-resolution measures of gaze and head behaviour that exposed compensatory strategies beyond binary success/failure. VR’s task parameters can be tuned (e.g., contrast and traffic density) while logging fine-grained gaze and head behaviour, which helps pinpoint the thresholds where deficits appear and tease apart sensory limits from task/cognitive demands [9]. The same platform supports training and rehabilitation: VR-based orientation-and-mobility instruction has transferred to safer real street-crossing in adults with severe vision loss [43], and immersive VR binocular therapy has produced improvements in acuity and stereopsis in older children with amblyopia [45].

### 4.5. Strengths and Limitations

Strengths: This study is one of the first to compare visual behaviour associated with central, peripheral, and combined vision impairment during the same dynamic, ecologically valid task. A reasonably sized cohort completed identical VR street-crossing and car-detection tasks, while a head-mounted eye tracker logged their gaze and head movements. Linking performance, i.e., detection success and time delay, with head- and eye-movement data, i.e., saccades, fixations, head turns, and exposed distinctive or absent compensation strategies for each impairment. Because the tasks replicate everyday safety hazards, measures like safe-crossing detection delay map directly onto real-world risk, while the broad severity of the visual impairment range revealed the impairment threshold, i.e., severe dual loss, where performance collapses, which is crucial for setting rehabilitation priorities.

Limitations: Peripheral and combined visual impairment samples were small, limiting statistical power, and disease etiology within each subgroup was heterogeneous. Patients were, on average, older than controls, so some response may reflect age rather than vision alone. Although VR provides strong experimental control, it remains a simulation: the absence of real danger, the daylight scene, and high-contrast vehicles may under- or over-estimate real-world difficulty. The tasks were limited (no low-contrast or night scenarios), and eye tracking also cannot guarantee that every fixation yielded visual awareness. Replication with larger, age-matched samples and broader task sets is warranted.

## 5. Conclusions

Using a head-mounted VR system with integrated head and eye tracking sensors, our study suggests that a combination of central and peripheral visual impairment presents the greatest challenge in dynamic traffic scenes, resulting in significant under-scanning and, in severe cases, total failure to detect targets. By contrast, peripheral visual impairment alone was associated with generally preserved exploratory behaviour, while central impairment tended to show shorter, more frequent saccades and relatively fewer head turns. Classifying patients by visual impairment type better explained task performance and visual behaviour than the WHO visual impairment classification, pointing toward the value of complementing VA/VF-based labels with functional descriptors. Although there were some limitations of this study—subgroups were small and heterogeneous, patients were older than controls, tasks were relatively simple (a controlled environment with no other pedestrians or noise disturbances)—we have shown that VR offers a safe and controllable platform on which to assess and potentially train functional vision, thereby bridging the gap between static clinical measures and the dynamic visual–motor demands of everyday navigation. Future work should recruit larger, age-matched cohorts; test diverse scenarios (traffic density, intersection complexity, adverse weather); add low-contrast and night-time conditions; and include real-world validation that compares VR-derived metrics with on-street performance and safety margins, building on evidence that mobility outcomes can be validated across real and virtual settings.

## Figures and Tables

**Table 1:** Categories of visual impairment according to WHO [3]. Abbreviations: pVA—presenting visual acuity (decimal); VF—visual field radius (degrees).

Category of Visual Impairment	pVA and VF Criteria	Number of Patients
Below normal but above threshold of WHO low-vision criteria	pVA ≤ 0.6 and ≥0.3 or VF < 80 and >20	12
1 (low vision)	pVA < 0.3 and ≥0.1	17
2 (low vision)	pVA < 0.1 and ≥0.05 or VF ≤ 20 and >10	3
3 (blindness)	pVA < 0.05 and ≥0.02 or VF ≤ 10 and >5	7
4 (blindness)	pVA < 0.02 and ≥light perception or VF ≤ 5	1

**Table 2:** Participants’ demographics; * inclusion criteria. Abbreviations: VA—uncorrected visual acuity (decimal); VF—visual field radius (degrees); MoCA—Montreal Cognitive Assessment, range 0–22; N/A—not applicable.

	Controls	Patients	Patients with Central Visual Impairment	Patients with Peripheral Visual Impairment	Patients with Combined Visual Impairment
N	19	40	26	6	8
Age (years), median [min–max]	50.0 [21.0–86.0]	71.5 [25.0–90.0]	78.5 [25.0–90.0]	55.5 [27.0–71.0]	56.5 [32.0–76.0]
Binocular uncorrected VA, median [min–max] (decimal/logMAR)	>0.8 */<0.10 *	0.30 [0.05–1.00]/0.52 [0.00–1.30]	0.30 [0.05–0.60]/0.52 [0.22–1.30]	0.85 [0.50–1.00]/0.07 [0.00–0.30]	0.25 [0.05–0.30]/0.61 [0.52–1.30]
Visual field radius (°), median [min–max]	>80 *	80 [5–80]	80 [60–80]	38 [10–50]	10 [5–35]
MoCA (0–22), median [min–max]	22 [20–22]	20 [15–22]	20 [15–22]	21 [20–21]	20 [17–22]
WHO categories of visual impairment	N/A				
Not categorized by WHO (n)		12	8	4	0
WHO category 1 (n)		17	16	0	1
WHO category 2 (n)		3	1	1	1
WHO category 3 (n)		7	1	1	5
WHO category 4 (n)		1	0	0	1

## Data Availability

Data is available from the corresponding author on reasonable request.

## Supplementary Materials

The following supporting information can be downloaded at: https://www.mdpi.com/article/10.3390/jemr18050055/s1. Table S1: Demographic characteristics, clinical data, and the results of crossing times and red car-counting tests in the patient group. VA—visual acuity; BCVA—best corrected visual acuity; VF—visual field; MoCA—Montreal Cognitive Assessment; AMD—age-related macular degeneration; RP—retinitis pigmentosa; STGD—Stargardt disease; LHH—left homonymous hemianopia; LHON—Leber hereditary optic neuropathy; ONA—optic nerve atrophy; MD—macular dystrophy; CM—chloroquine maculopathy; G—glaucoma; PDR—proliferative diabetic retinopathy; DME—diabetic macular edema; CRD—cone–rod dystrophy;/—no crossing decision made. Table S2: Demographic characteristics, clinical data, and the results of crossing times and red car-counting tests in the control group. MoCA—Montreal Cognitive Assessment;/— no crossing decision made. Table S3: Eye and head movements with *p*-values vs. controls (safe-crossing task). Median values and range are reported for each parameter. Statistical analysis comparing values between controls and patients was performed using two-sided Mann–Whitney tests. The significant *p*-values marking significant differences against controls are marked with asterisks. Table S4: Eye and head movements with *p*-values vs. controls (car-counting task). Median values and range are reported for each parameter. Statistical analysis comparing values between controls and patients was performed using two-sided Mann–Whitney tests. The significant *p*-values marking significant differences against controls are marked with asterisks. Figure S1: Average microsaccade velocity by task and visual impairment category. Boxplots: (**a**) safe-crossing task; (**b**) car-counting task. Boxes show the interquartile range (25th−75th percentiles); the line is the median; whiskers extend to 1.5x IQR; circles and stars denote outliers. Table S5. The results of eye and head movement metrics in the safe-crossing task in the patient group. S.—Saccades; C.—count; Avg.—average; A.—amplitude; V.—velocity; D—duration; N/A—not applicable/not available. Table S6. The results of eye and head movement metrics in the car-counting task in the patient group. S.—Saccades; C.—count; Avg.—average; A.—amplitude; V.—velocity; D—duration; N/A—not applicable/not available. Table S7. The results of eye and head movement metrics in the safe-crossing task in the control group. S.—Saccades; C.—count; Avg.—average; A.—amplitude; V.—velocity; D—duration. Table S8. The results of eye and head movement metrics in the car-counting task in the control group. S.—Saccades; C.—count; Avg.—average; A.—amplitude; V.—velocity; D—duration. Figure S2: Time points when participants thought it was safe to cross the street. Patients are further categorized by the WHO visual impairment categories. Figure S3: Saccade kinematics by WHO categories of visual impairment (safe-crossing task). Figure S4: Saccade kinematics by WHO categories of visual impairment (car-counting task). Figure S5: Number of head turns in correlation with the radius of the visual field in patients with peripheral visual impairment. Note the trend of more frequent head turns in patients with narrower visual fields. The correlation was not significant.
