# Supplementary material for: Head and Eye Movements During Pedestrian Crossing in Patients with Visual Impairment: A Virtual Reality Eye Tracking Study
Source: J Eye Mov Res. 2025 Oct 15;18(5):55. doi: 10.3390/jemr18050055 (PMC12565098; doi:10.3390/jemr18050055)
Supplement: Supplementary file 1 [file jemr-18-00055-s001.zip › jemr-3853927-supplementary.pdf]

Journal of *Eye Movement Research*

MDPI

Supplementary materials

Table S1. Demographic characteristics, clinical data, and the results of crossing times and red car-counting tests in the patient group.

ID	Age (years)	Diagnosis	Uncorrected VA	BCVA	Spherical equivalent in the better-seeing eye	VF radius (degrees)	Type of visual loss: central/ peripheral/ combined	Category of visual impairment: 0-4	MoCA Blind Test Score: 0-22	Crossing Time 1 (s)	Crossing Time 2 (s)	Counting red cars: 0-3
1	79	AMD	0.60	0.60	0,.5	80	central	0	18	19.40	38.63	3
2	87	AMD	0.30	0.40	-0.38	80	central	1	15	19.10	37.02	3
3	85	AMD	0.60	0.70	-0.50	80	central	0	17	19.10	42.22	3
4	86	AMD	0.30	0.30	0.38	80	central	1	20	19.75	37.38	3
5	83	AMD	0.50	0.70	-0.50	80	central	0	19	22.40	37.01	3
6	86	AMD	0.30	0.30	0.88	80	central	1	19	19.96	36.62	3
7	71	AMD	0.50	0.50	0.50	80	central	0	20	18.09	37.61	2
8	76	AMD	0.30	0.30	0.25	80	central	1	20	19.11	35.68	3
9	70	AMD	0.20	0.20	0.00	80	central	1	21	24.20	/	3
10	81	AMD	0.30	0.50	-1.00	80	central	1	19	19.86	37.99	3
11	76	AMD	0.30	0.30	0.00	70	central	1	18	16.79	38.06	3
12	89	AMD	0.40	0.40	-0.13	80	central	0	19	18.41	38.85	3
13	84	AMD	0.30	0.40	0.50	80	central	1	21	19.59	37.94	3
14	87	AMD	0.30	0.40	0.75	80	central	1	19	/	37.71	3
15	85	AMD	0.40	0.50	0.00	80	central	0	19	18.97	/	3
16	51	RP	0.80	1.00	0.13	15	peripheral	2	21	18.27	37.44	3
17	56	STGD	0.60	0.80	0.13	80	central	0	21	17.88	36.59	3
18	76	RP	0.30	0.40	0.00	20	combined	2	17	18.27	37.24	3
19	71	RP	0.90	1.00	-0.63	50	peripheral	0	20	18.68	38.04	3
20	27	LHH	1.00	1.00	-0.25	35	peripheral	0	21	19.63	37.16	2
21	49	LHON	0.20	0.20	0.50	5	combined	4	20	18.30	35.53	3
22	25	STGD	0.16	0.16	-1.63	80	central	1	22	15.55	34.41	3
23	37	RP	0.30	0.50	-1.00	10	combined	3	20	/	35.55	3
24	60	ONA	1.00	1.00	0.25	50	peripheral	0	20	21.30	38.03	3
25	38	MD	0.30	0.30	-0.38	80	central	1	19	16.70	34.96	3
26	51	MD	0.60	0.60	-0.88	80	central	0	21	17.20	34.79	3
27	43	STGD	0.08	0.10	-1.63	80	central	2	22	16.90	35.93	3
28	51	RP	0.50	0.70	0.00	40	peripheral	0	21	17.00	35.73	3
29	65	CM	0.05	0.08	1.25	80	central	3	21	19.10	36.92	3
30	64	G. PDR	0.16	0.16	0.00	35	combined	1	19	19.09	37.75	3
31	90	AMD	0.16	0.20	-0.25	80	central	1	21	20.65	38.81	3
32	77	AMD	0.16	0.20	0.00	80	central	1	19	20.50	35.40	3
33	78	AMD	0.20	0.20	-0.38	80	central	1	19	19.30	36.80	3
34	80	AMD	0.16	0.20	-0.25	80	central	1	20	20.00	39.30	2
35	72	DME	0.30	0.40	-1.13	60	central	1	20	22.00	37.50	3
36	32	CRD	0.05	0.16	-1.50	7	combined	3	21	/	/	0
37	42	RP	0.10	0.20	-1.13	7	combined	3	22	/	/	0

38	66	ONA	0.30	0.40	0.50	10	combined	3	20	20.30	38.80	3
39	74	RP	0.30	0.30	0.75	10	combined	3	19	20.52	38.20	3
40	70	RP	0.60	0.80	0.13	10	peripheral	3	21	/	36.70	3

VA—visual acuity; BCVA—best corrected visual acuity; VF—visual field; MoCA—Montreal Cognitive Assessment; AMD—age-related macular degeneration; RP—retinitis pigmentosa; STGD—Stargardt disease; LHH—left homonymous hemianopia; LHON—Leber hereditary optic neuropathy; ONA—optic nerve atrophy; MD—macular dystrophy; CM—chloroquine maculopathy; G—glaucoma; PDR—proliferative diabetic retinopathy; DME—diabetic macular edema; CRD—cone–rod dystrophy; /—no crossing decision made

Table S2. Demographic characteristics, clinical data, and the results of crossing times and red car-counting tests in the control group.

ID	Age (years)	MoCA Blind Test Score: 0-22	Crossing Time 1	Crossing Time 2	Counting Red Cars: 0-3
1	46	22	17.82	35.79	3
2	47	21	17.60	36.73	3
3	25	22	/	35.59	3
4	74	20	16.58	35.33	3
5	46	22	18.46	36.14	3
6	30	22	17.32	36.85	3
7	52	22	15.85	35.27	3
8	50	21	17.52	35.23	3
9	47	22	/	35.95	3
10	49	22	19.90	37.29	3
11	73	22	/	35.32	3
12	63	22	/	35.78	3
13	38	22	18.13	35.28	3
14	60	21	17.95	36.30	3
15	57	22	18.28	35.86	3
16	69	22	19.52	37.76	3
17	21	21	17.80	36.52	3
18	56	21	17.70	35.22	3
19	86	20	16.70	35.77	3

MoCA—Montreal Cognitive Assessment; /— no crossing decision made.

Table S3. Eye and head movements with p-values vs. controls (safe-crossing task).

**Metric**	**Controls**	**Central loss**	**Peripheral loss**	**Combined loss**	**All patients**
**Number of head turns**	**19 [8-40]**	16 [7-56]; p=0.134	20 [2-44]; p=0.899	12 [2-25]; **p=0.049***	16 [2-56]; p=0.104
Total saccades (n)	326 [243-419]	328 [164-439]; p=0.748	291 [223-350]; p=0.611	269 [142-428]; p=0.203	320 [142-439]; p=0.791
Microsaccades (n)	103 [61-186]	108 [22-189]; p=0.428	104 [22-125]; p=0.464	78 [24-195]; p=0.165	99 [22-195]; p=0.239

**Macrosaccades (n)**	**195 [156-256]**	226 [121-278]; **p=0.033***	195 [165-253]; p=0.824	190 [118-233]; p=0.452	212 [118-278]; p=0.211
Total saccade duration (s)	2.635 [1.965-3.447]	2.638 [1.383-3.346]; p=0.918	2.328 [1.926-2.835]; p=0.475	2.183 [1.184-3.259]; p=0.188	2.604 [1.184-3.346]; p=0.619
Average saccade duration (s)	0.008 [0.008-0.009]	0.008 [0.008-0.008]; p=0.918	0.008 [0.008-0.009]; p=0.828	0.008 [0.008-0.008]; p=0.735	0.008 [0.008-0.009]; p=0.974
Total microsaccade duration (s)	0.513 [0.263-0.930]	0.478 [0.067-1.059]; p=0.455	0.508 [0.090-0.607]; p=0.555	0.295 [0.121-0.855]; p=0.107	0.478 [0.067-1.059]; p=0.239
Average microsaccade duration (s)	0.005 [0.004-0.006]	0.005 [0.003-0.006]; p=0.973	0.005 [0.004-0.005]; p=0.975	0.004 [0.002-0.006]; p=0.534	0.005 [0.002-0.006]; p=0.868
**Total macrosaccade duration (s)**	**1.604 [1.272-2.093]**	1.846 [1.028-2.252]; **p=0.044***	1.654 [1.313-2.076]; p=0.828	1.570 [0.986-1.835]; p=0.461	1.748 [0.986-2.252]; p=0.246
Average macrosaccade duration (s)	0.008 [0.008-0.009]	0.008 [0.008-0.008]; p=0.899	0.008 [0.008-0.009]; p=0.687	0.008 [0.008-0.008]; p=0.910	0.008 [0.008-0.009]; p=0.987
Average saccade velocity (°/s)	1213.0 [831.2-2636.4]	1160.1 [660.3-3077.5]; p=0.722	1355.8 [896.6-2883.9]; p=0.333	1184.9 [656.0-2382.6]; p=0.461	1187.4 [656.0-3077.5]; p=0.829
**Average microsaccade velocity (°/s)**	**115.2 [108.1-129.4]**	118.8 [108.6-144.0]; **p=0.019***	117.0 [110.5-128.2]; p=0.274	124.3 [103.9-195.1]; p=0.169	118.8 [103.9-195.1]; **p=0.016***
Average macrosaccade velocity (°/s)	1817.9 [1201.6-4044.6]	1626.9 [988.7-3520.1]; p=0.317	2073.5 [1311.3-3187.5]; p=0.555	1508.1 [1123.8-2841.4]; p=0.083	1686.6 [988.7-3520.1]; p=0.289
**Total saccade amplitude (°)**	**3147.0 [2293.0-5180.0]**	3042.0 [1579.0-6833.0]; p=0.973	3154.5 [2340.0-5554.0]; p=0.828	2388.0 [1796.0-2820.0]; **p=0.006***	2935.0 [1579.0-6833.0]; p=0.508
Average saccade amplitude (°)	9.81 [6.89-21.32]	9.48 [5.18-25.40]; p=0.705	11.05 [7.27-24.91]; p=0.400	9.36 [5.08-19.86]; p=0.534	9.77 [5.08-25.40]; p=0.817
Total microsaccade amplitude (°)	58.0 [32.0-103.0]	56.0 [8.0-127.0]; p=0.581	59.5 [10.0-74.0]; p=0.656	38.0 [17.0-93.0]; p=0.148	54.0 [8.0-127.0]; p=0.345
**Total macrosaccade amplitude (°)**	**3098.0 [2244.0-5146.0]**	2970.0 [1521.0-6815.0]; p=0.973	3089.0 [2278.0-5544.0]; p=0.828	2353.0 [1716.0-2802.0]; **p=0.008***	2861.0 [1521.0-6815.0]; p=0.487
Average microsaccade amplitude (°)	0.53 [0.39-0.65]	0.54 [0.36-0.70]; p=0.462	0.54 [0.45-0.63]; p=0.877	0.51 [0.46-0.75]; p=0.955	0.53 [0.36-0.75]; p=0.545
Average macrosaccade amplitude (°)	14.72 [9.80-32.99]	13.32 [8.00-29.12]; p=0.352	17.09 [10.85-27.58]; p=0.475	12.58 [8.93-23.75]; p=0.094	13.51 [8.00-29.12]; p=0.337
**Average fixation duration (s)**	**0.231 [0.176-0.288]**	0.200 [0.162-0.372]; **p=0.033***	0.231 [0.178-0.273]; p=0.824	0.237 [0.193-0.381]; p=0.452	0.212 [0.162-0.381]; p=0.211

Median values and range are reported for each parameter. Statistical analysis comparing values between controls and patients was performed using two-sided Mann–Whitney tests. The significant p-values marking significant differences against controls are marked with asterisks.

Table S4. Eye and head movements with p-values vs. controls (car-counting task).

Metric	Controls	Central loss	Peripheral loss	Combined loss	All patients
**Head turns (n)**	**35 [18-57]**	29 [7-48]; p=0.069	39 [25-112]; p=0.545	20 [2-45]; **p=0.036***	29 [2-112]; p=0.077
Total saccades (n)	314 [237-392]	295 [199-427]; p=0.323	309 [223-356]; p=0.687	284 [142-377]; p=0.214	294 [142-427]; p=0.233
**Microsaccades (n)**	**119 [73-183]**	86 [21-164]; **p=0.002***	109 [22-130]; p=0.340	65 [24-130]; **p=0.022***	89 [21-164]; **p=0.001***
Macrosaccades (n)	196 [142-241]	208 [112-279]; p=0.089	198 [180-244]; p=0.484	204 [118-257]; p=0.862	207 [112-279]; p=0.140
Total saccade duration (s)	2.516 [1.870-3.174]	2.354 [1.540-3.348]; p=0.138	2.533 [1.926-2.846]; p=0.877	2.214 [1.184-2.914]; p=0.135	2.344 [1.184-3.348]; p=0.119
Average saccade duration (s)	0.008 [0.008-0.008]	0.008 [0.007-0.009]; p=0.364	0.008 [0.008-0.009]; p=0.333	0.008 [0.008-0.009]; p=0.821	0.008 [0.007-0.009]; p=0.753
**Total microsaccade duration (s)**	**0.527 [0.299-0.917]**	0.370 [0.078-0.844]; **p=0.009***	0.549 [0.090-0.636]; p=0.733	0.233 [0.142-0.618]; **p=0.022***	0.365 [0.078-0.844]; **p=0.008***
Average microsaccade duration (s)	0.005 [0.003-0.005]	0.004 [0.003-0.006]; p=0.455	0.005 [0.004-0.006]; p=0.828	0.004 [0.003-0.006]; p=0.955	0.004 [0.003-0.006]; p=0.619
Total macrosaccade duration (s)	1.625 [1.172-1.992]	1.708 [0.881-2.275]; p=0.210	1.659 [1.504-1.996]; p=0.475	1.777 [0.986-2.111]; p=0.778	1.710 [0.881-2.275]; p=0.233
Average macrosaccade duration (s)	0.008 [0.008-0.009]	0.008 [0.008-0.009]; p=0.145	0.008 [0.008-0.009]; p=0.514	0.008 [0.008-0.009]; p=0.821	0.008 [0.008-0.009]; p=0.345
Average saccade velocity (°/s)	1123.9 [658.3-2072.3]	1138.0 [573.6-4105.0]; p=0.899	1160.8 [938.4-2883.9]; p=0.642	1188.6 [641.0-2382.6]; p=0.692	1164.6 [573.6-4105.0]; p=0.728
Average microsaccade velocity (°/s)	116.7 [107.2-132.5]	120.1 [108.7-164.4]; p=0.064	117.7 [107.1-145.0]; p=1.000	124.6 [113.1-193.9]; p=0.073	120.0 [107.1-193.9]; p=0.064
Average macrosaccade velocity (°/s)	1718.5 [937.6-3536.7]	1574.6 [792.0-4539.1]; p=0.415	1728.5 [1411.2-3187.5]; p=0.877	1543.6 [1080.9-2841.4]; p=0.427	1650.2 [792.0-4539.1]; p=0.436
Total Saccade amplitude (°)	2716.0 [1942.0-4822.0]	2533.0 [1029.0-7272.0]; p=0.688	2940.0 [2482.0-5554.0]; p=0.303	2581.0 [1384.0-3875.0]; p=0.461	2641.0 [1029.0-7272.0]; p=0.817

Average saccade amplitude (°)	9.06 [5.26-16.61]	9.03 [4.22-33.98]; p=0.936	9.52 [7.50-24.91]; p=0.642	10.06 [4.87-19.86]; p=0.651	9.24 [4.22-33.98]; p=0.740
**Total microsaccade amplitude (°)**	**60.0 [33.0-107.0]**	48.0 [9.0-94.0]; **p=0.012***	63.0 [10.0-74.0]; p=0.824	36.0 [18.0-74.0]; **p=0.024***	48.0 [9.0-94.0]; **p=0.011***
Total macrosaccade amplitude (°)	2663.0 [1865.0-4715.0]	2485.0 [988.0-7263.0]; p=0.705	2877.0 [2412.0-5544.0]; p=0.366	2552.0 [1310.0-3835.0]; p=0.572	2597.0 [988.0-7263.0]; p=0.842
Average microsaccade amplitude (°)	0.56 [0.35-0.61]	0.50 [0.42-0.77]; p=0.792	0.56 [0.46-0.68]; p=0.588	0.55 [0.49-0.75]; p=0.772	0.53 [0.42-0.77]; p=0.667
Average macrosaccade amplitude (°)	13.94 [7.74-28.80]	12.68 [6.06-37.63]; p=0.402	14.03 [11.49-27.58]; p=0.780	13.22 [8.51-23.75]; p=0.497	13.65 [6.06-37.63]; p=0.466
Average fixation duration (s)	0.230 [0.187-0.317]	0.217 [0.161-0.402]; p=0.089	0.227 [0.184-0.250]; p=0.484	0.221 [0.175-0.381]; p=0.862	0.217 [0.161-0.402]; p=0.140

Median values and range are reported for each parameter. Statistical analysis comparing values between controls and patients was performed using two-sided Mann–Whitney tests. The significant p-values marking significant differences against controls are marked with asterisks.

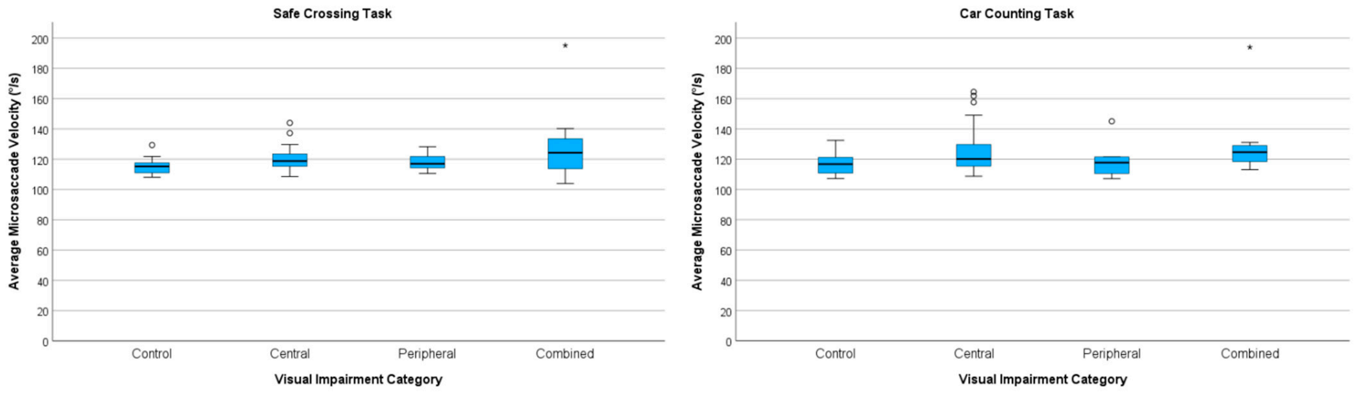

(a) **(b)**

Figure S1. Average microsaccade velocity by task and visual impairment category. Boxplots: (**a**) safe-crossing task; (**b**) car-counting task. Boxes show the interquartile range (25th–75th percentiles); the line is the median; whiskers extend to 1.5x IQR; circles and stars denote outliers.

Table S5. The results of eye and head movement metrics in the safe-crossing task in the patient group.

ID	S. C. - micro (n)	S. C. - macro (n)	S. C. - total (n)	Avg. S. A. - micro (°)	Avg. S. A. - macro (°)	Combined S. A. - total (°)	Avg. S. V. - micro (°/s)	Avg. S. V. - macro (°/s)	Avg. S. D. - micro (s)	Avg. S. D. - macro (s)	Total S. D. - micro (s)	Total S. D. - macro (s)	Total S. D. (s)	Avg. fixation D. (s)	Head turns C. - total (n)
1	135	224	359	0.51	8.00	1861	115.27	988.72	0.00443	0.00809	0.60	1.81	20.90	0.20	18
2	35	234	269	0.51	29.12	6833	118.29	3520.13	0.00435	0.00827	0.15	1.94	42.32	0.19	19
3	99	234	333	0.51	14.34	3406	116.63	1783.83	0.00433	0.00804	0.43	1.88	29.22	0.19	12
4	71	247	318	0.70	22.13	5515	126.01	2690.05	0.00559	0.00823	0.40	2.03	37.39	0.18	15
5	22	212	234	0.36	22.85	4852	119.18	2739.93	0.00305	0.00834	0.07	1.77	28.47	0.21	7
6	188	207	395	0.68	13.14	2847	119.88	1567.17	0.00564	0.00838	1.06	1.74	22.55	0.22	9
7	116	233	349	0.54	14.94	3544	110.94	1818.36	0.00489	0.00822	0.57	1.91	26.49	0.19	23
8	111	212	323	0.56	12.53	2719	117.36	1499.13	0.00476	0.00836	0.53	1.77	23.65	0.21	19
9	122	222	344	0.49	13.51	3059	125.21	1686.59	0.00393	0.00801	0.48	1.78	24.24	0.20	10
10	107	247	354	0.63	15.08	3792	129.72	1796.22	0.00483	0.00840	0.52	2.07	27.95	0.18	35
11	189	250	439	0.59	9.21	2415	116.22	1179.46	0.00510	0.00781	0.96	1.95	23.82	0.18	7
12	128	243	371	0.46	10.32	2567	109.81	1334.74	0.00420	0.00773	0.54	1.88	23.64	0.19	12
13	108	223	331	0.49	10.81	2464	114.05	1378.91	0.00431	0.00784	0.46	1.75	23.02	0.20	16
14	132	278	410	0.64	10.58	3025	118.78	1305.99	0.00535	0.00810	0.71	2.25	28.38	0.16	15
15	43	267	310	0.70	16.76	4504	137.23	2028.76	0.00509	0.00826	0.22	2.21	36.95	0.17	17
16	22	201	223	0.46	27.58	5554	110.55	3187.45	0.00412	0.00865	0.09	1.74	26.58	0.22	44
17	109	140	249	0.53	10.86	1579	110.99	1357.12	0.00479	0.00801	0.52	1.12	13.36	0.32	16
18	78	200	278	0.53	12.80	2601	124.27	1508.11	0.00423	0.00849	0.33	1.70	19.62	0.23	25
19	97	253	350	0.45	17.54	4481	128.23	2137.22	0.00354	0.00821	0.34	2.08	30.96	0.18	2
20	120	172	292	0.62	16.63	2935	121.86	2036.23	0.00506	0.00817	0.61	1.41	20.78	0.26	11
21	69	187	256	0.51	12.58	2388	118.57	1578.42	0.00428	0.00797	0.30	1.49	20.41	0.24	9
22	43	121	164	0.44	28.55	3474	120.86	3362.03	0.00366	0.00849	0.16	1.03	16.69	0.37	11
23	161	190	351	0.50	9.03	1796	103.91	1123.75	0.00478	0.00804	0.77	1.53	18.28	0.24	17
24	125	165	290	0.52	14.13	2396	114.20	1775.74	0.00455	0.00796	0.57	1.31	19.92	0.27	20
25	132	171	303	0.53	16.90	2960	115.11	2035.55	0.00460	0.00830	0.61	1.42	22.82	0.26	15
26	72	175	247	0.50	19.84	3508	126.75	2400.71	0.00394	0.00826	0.28	1.45	23.47	0.26	20
27	92	251	343	0.57	18.47	4689	121.45	2224.35	0.00465	0.00831	0.43	2.08	31.05	0.18	23
28	91	189	280	0.63	17.55	3374	115.09	2110.77	0.00544	0.00831	0.50	1.57	22.39	0.24	21
29	65	165	230	0.52	12.59	2112	143.97	1485.87	0.00363	0.00848	0.24	1.40	16.62	0.27	56
30	24	118	142	0.75	23.75	2820	126.83	2841.41	0.00591	0.00836	0.14	0 .99	17.87	0.38	20
31	81	243	324	0.57	12.84	3166	118.80	1543.56	0.00478	0.00832	0.39	2.02	27.25	0.19	21
32	122	209	331	0.62	10.72	2316	121.73	1327.42	0.00505	0.00808	0.62	1.69	20.91	0.22	21
33	91	192	283	0.58	11.21	2205	116.65	1341.51	0.00499	0.00835	0.45	1.60	19.68	0.23	21
34	115	250	365	0.47	11.72	2985	108.57	1482.97	0.00433	0.00791	0.50	1.98	23.85	0.18	13
35	93	227	320	0.63	17.35	3997	123.55	2090.40	0.00513	0.00830	0.48	1.88	30.18	0.20	23
36	25	191	216	0.68	11.04	2126	140.24	1328.37	0.00485	0.00831	0.12	1.59	21.57	0.24	3
37	82	187	269	0.46	14.85	2814	195.12	1768.57	0.00237	0.00839	0.19	1.57	15.76	0.24	6
38	N/A	N/A	N/A	N/A	N/A	N/A	N/A	N/A	N/A	N/A	N/A	N/A	N/A	N/A	16
39	195	233	428	0.48	8.93	2174	108.76	1134.32	0.00439	0.00787	0.86	1.83	22.44	0.19	2
40	112	210	322	0.55	10.85	2340	118.93	1311.30	0.00466	0.00827	0.52	1.74	21.64	0.21	29

S.—Saccades; C.—count; Avg.—average; A.—amplitude; V.—velocity; D—duration; N/A—not applicable/not available.

Table S6. The results of eye and head movement metrics in the car-counting task in the patient group.

ID	S. C. - micro (n)	S. C. - macro (n)	S. C. - total (n)	Avg. S. A. - micro (°)	Avg. S. A. - macro (°)	Combined S. A. - total (°)	Avg. S. V. - micro (°/s)	Avg. S. V. - macro (°/s)	Avg. S. D.- micro (s)	Avg. S. D. - macro (s)	Total S. D. - micro (s)	Total S. D. - macro (s)	Total S. D. (s)	Avg. fixation D. (s)	Head turns C. - total (n)
1	99	204	303	0.42	6.06	1278	139.35	792.00	0.00304	0.007650	0.30	1.56	14.27	0.22	28
2	21	193	214	0.43	37.63	7272	115.29	4539.08	0.00372	0.008291	0.08	1.60	42.65	0.23	45
3	103	226	329	0.47	9.84	2271	119.90	1261.67	0.00389	0.007796	0.40	1.76	22.37	0.20	12
4	83	244	327	0.60	25.63	6304	113.76	3091.81	0.00529	0.008290	0.44	2.02	38.08	0.18	33
5	37	207	244	0.60	19.07	3969	149.08	2308.23	0.00399	0.008261	0.15	1.71	28.26	0.22	20
6	135	207	342	0.57	8.73	1883	121.93	1078.71	0.00467	0.008088	0.63	1.67	18.99	0.22	9
7	164	263	427	0.57	9.13	2495	111.33	1140.65	0.00515	0.008003	0.84	2.10	24.60	0.17	39
8	122	236	358	0.54	11.25	2721	120.24	1366.94	0.00450	0.008230	0.55	1.94	24.66	0.19	33
9	79	208	287	0.43	13.65	2874	108.99	1650.18	0.00395	0.008274	0.31	1.72	19.61	0.22	27
10	75	141	216	0.47	13.76	1975	157.61	1700.17	0.00296	0.008093	0.22	1.14	12.30	0.32	48
11	102	182	284	0.49	7.96	1499	117.86	1040.29	0.00416	0.007654	0.42	1.39	15.62	0.25	7
12	62	207	269	0.42	9.01	1892	120.50	1186.40	0.00348	0.007598	0.22	1.57	18.65	0.22	14
13	60	215	275	0.42	11.70	2540	115.42	1498.96	0.00361	0.007804	0.22	1.68	21.92	0.21	17
14	108	225	333	0.62	6.26	1476	117.82	792.42	0.00526	0.007902	0.57	1.78	18.50	0.20	15
15	65	279	344	0.77	14.02	3962	137.00	1719.30	0.00561	0.008156	0.36	2.28	34.38	0.16	34
16	22	201	223	0.46	27.58	5554	110.55	3187.45	0.00412	0.008653	0.09	1.74	26.58	0.22	44
17	93	112	205	0.44	8.82	1029	121.82	1121.69	0.00362	0.007864	0.34	0.88	10.60	0.40	25
18	52	157	209	0.52	13.22	2102	117.04	1543.56	0.00443	0.008563	0.23	1.34	15.28	0.29	45
19	92	244	336	0.57	13.68	3391	145.04	1673.16	0.00390	0.008179	0.36	2.00	25.32	0.18	112
20	127	180	307	0.55	16.32	3007	119.03	1890.65	0.00463	0.008630	0.59	1.55	21.58	0.25	25
21	65	228	293	0.55	13.97	3222	131.11	1737.61	0.00423	0.008042	0.27	1.83	27.84	0.20	19
22	48	206	254	0.48	22.47	4652	129.73	2714.49	0.00369	0.008278	0.18	1.71	28.22	0.22	23
23	130	154	284	0.57	8.51	1384	119.79	1080.95	0.00475	0.007869	0.62	1.21	14.50	0.29	21
24	115	196	311	0.49	14.37	2873	107.15	1783.75	0.00455	0.008057	0.52	1.58	24.51	0.23	38
25	105	176	281	0.47	13.95	2504	111.64	1707.88	0.00418	0.008167	0.44	1.44	20.72	0.26	26
26	92	202	294	0.52	21.71	4434	128.07	2614.69	0.00408	0.008304	0.37	1.68	27.86	0.22	38
27	89	245	334	0.62	19.27	4776	119.75	2286.32	0.00516	0.008428	0.46	2.06	31.41	0.18	44
28	103	181	284	0.68	13.33	2482	121.46	1603.66	0.00560	0.008310	0.58	1.50	19.48	0.25	40
29	28	171	199	0.68	15.33	2641	164.43	1743.29	0.00413	0.008795	0.12	1.50	16.92	0.26	38
30	24	118	142	0.75	23.75	2820	126.83	2841.41	0.00591	0.008357	0.14	0.99	17.87	0.38	42
31	81	215	296	0.67	10.39	2288	120.03	1298.81	0.00556	0.008000	0.45	1.72	20.41	0.21	22
32	64	244	308	0.42	20.13	4938	161.81	2470.66	0.00261	0.008146	0.17	1.99	34.57	0.18	31
33	109	183	292	0.50	10.73	2017	108.70	1296.68	0.00455	0.008273	0.50	1.51	19.66	0.25	45
34	122	240	362	0.60	10.22	2526	117.77	1264.30	0.00508	0.008084	0.62	1.94	22.46	0.19	30
35	59	245	304	0.51	21.85	5383	120.27	2606.91	0.00422	0.008381	0.25	2.05	37.45	0.18	47
36	53	204	257	0.55	12.51	2581	124.62	1436.00	0.00439	0.008712	0.23	1.78	23.39	0.22	15
37	81	257	338	0.49	14.92	3875	193.94	1817.08	0.00255	0.008212	0.21	2.11	22.40	0.18	9
38	N/A	N/A	N/A	N/A	N/A	N/A	N/A	N/A	N/A	N/A	N/A	N/A	N/A	N/A	29
39	127	250	377	0.53	8.86	2283	113.11	1124.04	0.00467	0.007886	0.59	1.97	21.32	0.18	2
40	130	226	356	0.57	11.49	2671	116.41	1411.18	0.00489	0.008143	0.64	1.84	23.39	0.20	29

S.—Saccades; C.—count; Avg.—average; A.—amplitude; V.—velocity; D—duration; N/A—not applicable/not available.

Table S7. The results of eye and head movement metrics in the safe-crossing task in the control group.

ID	S. C. - micro (n)	S. C. - macro (n)	S. C. - total (n)	Avg. S. A. - micro (°)	Avg. S. A. - macro (°)	Combined S. A. - total (°)	Avg. S. V. - micro (°/s)	Avg. S. V. - macro (°/s)	Avg. S. D. - micro (s)	Avg. S. D. - macro (s)	Total S. D. - micro (s)	Total S. D. - macro (s)	Total S. D. (s)	Avg. fixation D. (s)	Head turns C. - total (n)
1	98	179	277	0.50	17.31	3147	118.52	2147.19	0.00422	0.00806	0.41	1.44	23.12	0.25	24
2	127	230	357	0.65	14.71	3466	117.57	1817.90	0.00556	0.00809	0.71	1.86	28.24	0.20	8
3	151	176	327	0.44	13.05	2362	111.68	1569.94	0.00391	0.00831	0.59	1.46	18.15	0.26	16
4	125	223	348	0.50	10.47	2397	115.78	1232.18	0.00428	0.00850	0.54	1.90	21.93	0.20	21
5	131	204	335	0.47	18.00	3734	108.10	2227.83	0.00438	0.00808	0.57	1.65	26.71	0.22	15
6	81	168	249	0.54	13.39	2293	110.47	1627.67	0.00492	0.00822	0.40	1.38	17.91	0.27	24
7	77	203	280	0.62	15.40	3174	129.35	1861.55	0.00482	0.00827	0.37	1.68	24.42	0.22	15
8	182	237	419	0.57	13.90	3397	110.81	1657.31	0.00511	0.00839	0.93	1.99	25.11	0.19	19
9	155	187	342	0.50	20.29	3872	109.97	2458.56	0.00457	0.00825	0.71	1.54	22.21	0.24	16
10	131	195	326	0.58	16.51	3295	111.72	2006.34	0.00519	0.00823	0.68	1.60	23.16	0.23	30
11	88	198	286	0.44	12.73	2560	116.73	1588.99	0.00380	0.00801	0.33	1.59	21.30	0.23	14
12	186	193	379	0.50	18.79	3719	108.89	2320.55	0.00455	0.00810	0.84	1.56	23.51	0.23	19
13	61	208	269	0.53	20.60	4317	121.82	2433.55	0.00431	0.00847	0.26	1.76	26.23	0.22	20
14	91	188	279	0.58	13.60	2610	116.87	1646.77	0.00498	0.00826	0.45	1.55	20.32	0.24	18
15	93	195	288	0.53	14.72	2919	115.25	1638.69	0.00457	0.00898	0.43	1.75	21.25	0.23	20
16	121	256	377	0.60	11.65	3053	118.74	1423.98	0.00501	0.00818	0.61	2.09	26.16	0.18	27
17	87	156	243	0.39	32.99	5180	111.15	4044.65	0.00352	0.00816	0.31	1.27	27.93	0.29	17
18	92	177	269	0.59	16.46	2967	117.72	2043.84	0.00499	0.00805	0.46	1.43	22.56	0.25	31
19	103	229	332	0.56	9.80	2302	113.06	1201.64	0.00498	0.00815	0.51	1.87	23.39	0.20	40

S.—Saccades; C.—count; Avg.—average; A.—amplitude; V.—velocity; D—duration.

Table S8. The results of eye and head movement metrics in the car-counting task in the control group.

ID	S. C. - micro (n)	S. C. - macro (n)	S. C. - total (n)	Avg. S. A. - micro (°)	Avg. S. A. - macro (°)	Combined S. A. - total (°)	Avg. S. V. - micro (°/s)	Avg. S. V. - macro (°/s)	Avg. S. D.- micro (s)	Avg. S. D. - macro (s)	Total S. D. - micro (s)	Total S. D. - macro (s)	Total S. D. (s)	Avg. fixation D. (s)	Head turns C. - total (n)
1	91	146	237	0.48	16.87	2507	119.66	2101.73	0.00404	0.008027	0.37	1.17	18.150	0.31	37
2	160	219	379	0.56	13.94	3142	119.32	1718.48	0.00466	0.008112	0.75	1.78	25.982	0.21	18
3	133	142	275	0.40	18.75	2716	109.11	2217.13	0.00365	0.008459	0.49	1.20	17.784	0.32	39
4	128	241	369	0.60	7.74	1942	132.49	937.63	0.00454	0.008254	0.58	1.99	20.459	0.19	28
5	115	211	326	0.46	12.37	2662	107.20	1534.97	0.00430	0.008056	0.49	1.70	23.264	0.21	28
6	81	168	249	0.54	13.39	2293	110.47	1627.67	0.00492	0.008225	0.40	1.38	17.909	0.27	24
7	73	194	267	0.56	17.20	3377	131.78	2078.98	0.00426	0.008271	0.31	1.60	25.206	0.23	29
8	183	199	382	0.57	10.60	2214	118.28	1297.49	0.00485	0.008168	0.89	1.63	20.058	0.23	36
9	148	241	389	0.57	16.96	4172	110.85	2059.14	0.00512	0.008238	0.76	1.99	26.270	0.19	33
10	151	189	340	0.57	11.35	2231	116.12	1398.07	0.00491	0.008118	0.74	1.53	19.638	0.24	46
11	121	193	314	0.56	14.30	2828	113.31	1757.54	0.00496	0.008137	0.60	1.57	22.871	0.23	27
12	180	212	392	0.59	22.24	4822	116.74	2710.41	0.00509	0.008206	0.92	1.74	32.154	0.21	38
13	93	172	265	0.36	16.28	2833	110.29	1947.37	0.00322	0.008359	0.30	1.44	19.891	0.26	31
14	80	196	276	0.54	16.68	3312	124.78	2000.92	0.00431	0.008336	0.34	1.63	24.260	0.23	35

15	93	198	291	0.40	13.14	2638	111.97	1524.61	0.00355	0.008616	0.33	1.71	20.356	0.23	48
16	112	238	350	0.56	12.46	3028	119.50	1488.50	0.00471	0.008369	0.53	1.99	25.660	0.19	45
17	119	158	277	0.42	28.80	4601	128.03	3536.75	0.00328	0.008144	0.39	1.29	24.825	0.28	30
18	128	162	290	0.47	13.31	2216	110.68	1646.20	0.00424	0.008085	0.54	1.31	18.993	0.28	50
19	117	221	338	0.62	9.36	2140	122.88	1128.44	0.00500	0.008292	0.59	1.83	21.623	0.20	57

S.—Saccades; C.—count; Avg.—average; A.—amplitude; V.—velocity; D—duration.

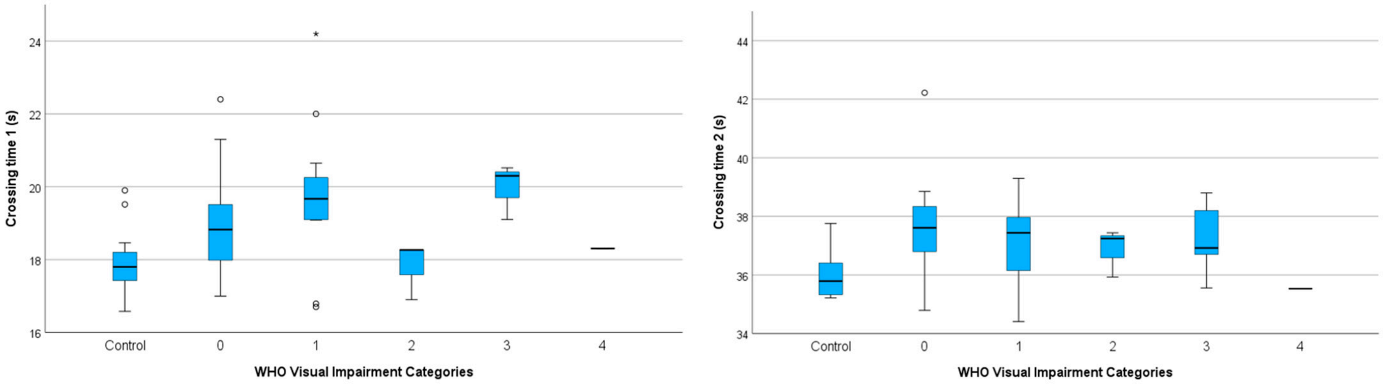

Figure S2. Time points when participants thought it was safe to cross the street. Patients are further categorized by the WHO visual impairment categories.

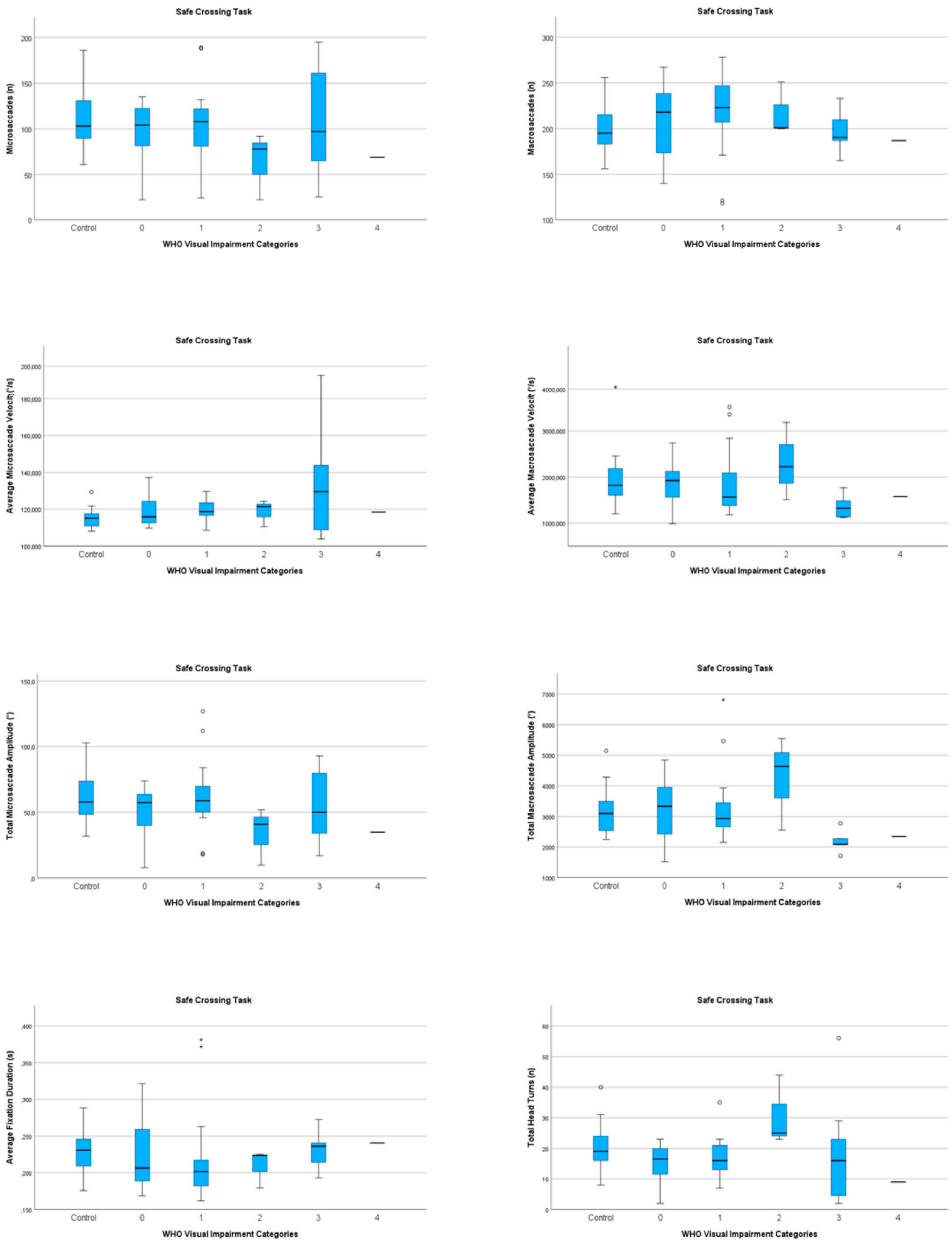

Figure S3. Saccade kinematics by WHO categories of visual impairment (safe-crossing task).

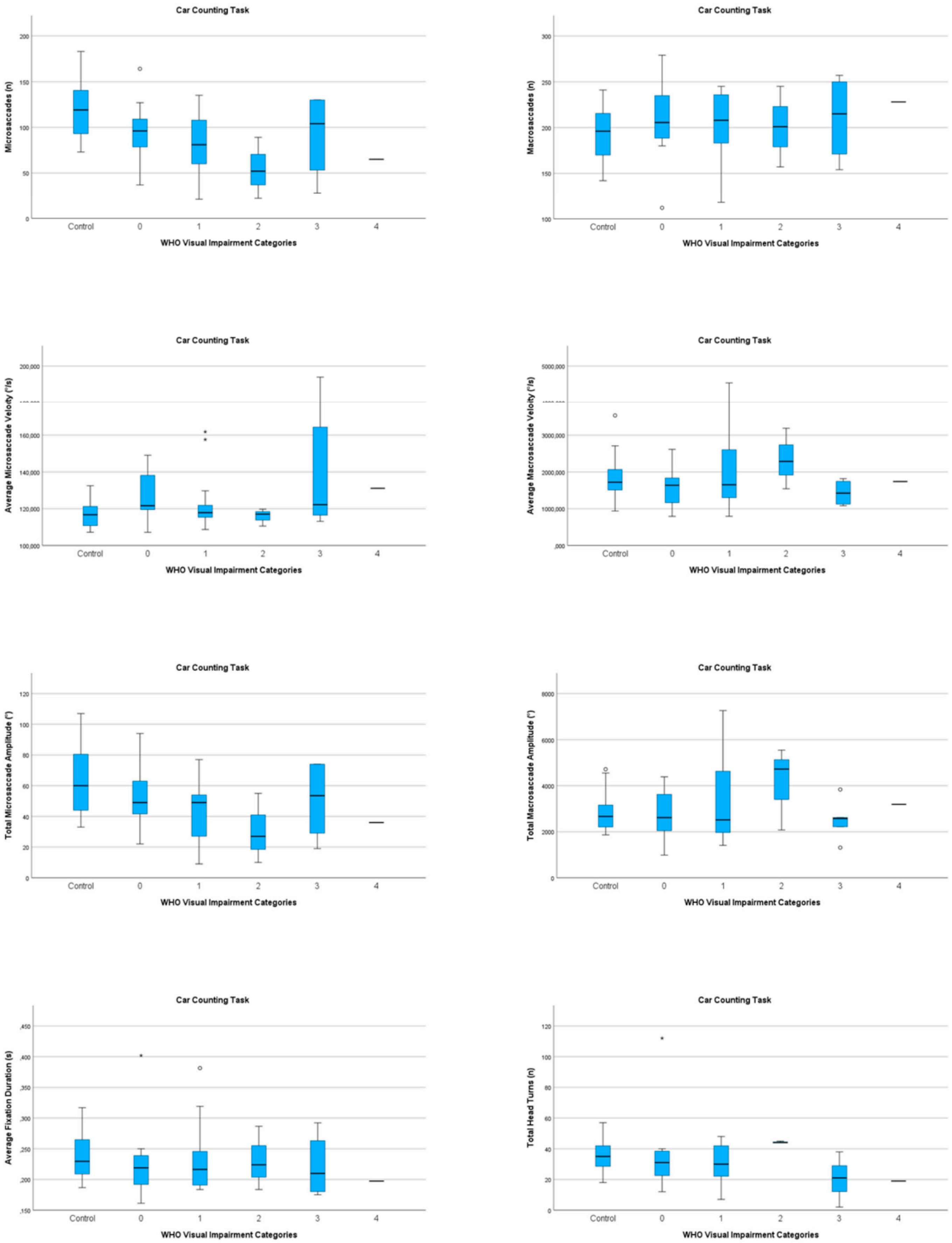

Figure S4. Saccade kinematics by WHO categories of visual impairment (car-counting task).

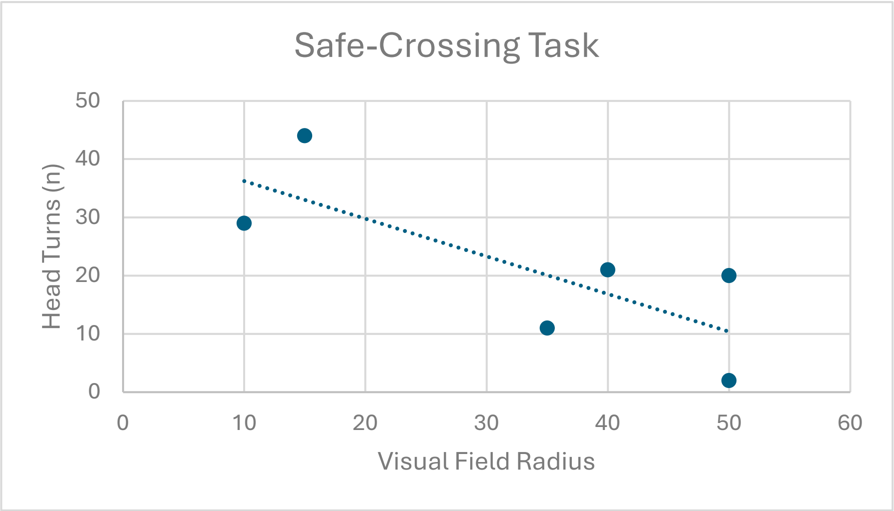

Figure S5. Number of head turns in correlation with the radius of the visual field in patients with peripheral visual impairment. Note the trend of more frequent head turns in patients with narrower visual fields. The correlation was not significant.
